# Endometrial Histopathology in Patients with Laparoscopic Proven Salpingitis and HIV-1 Infection

**DOI:** 10.1155/2011/407057

**Published:** 2011-09-20

**Authors:** Nelly R. Mugo, Julia Kiehlbauch, Nancy Kiviat, Rosemary Nguti, Joseph W. Gichuhi, Walter E. Stamm, Craig R. Cohen

**Affiliations:** ^1^Department of Obstetrics and Gynecology, Kenyatta National Hospital, P.O. Box 19865-00202, Nairobi, Kenya; ^2^Centre for Microbiology Research, Kenya Medical Research Institute, Nairobi, Kenya; ^3^Department of Pathology, Harborview Medical Center, UW Medicine Pathology, University of Washington, P.O. Box 357470, Seattle, WA 98195-7470, USA; ^4^Maryland Department of Health and Mental Hygiene, 201 West Preston Street, Baltimore, MD 21201, USA; ^5^Department of Statistics, University of Nairobi, Nairobi, Kenya; ^6^Department of Obstetrics and Gynecology, University of Nairobi, School of Medicine, Kenyatta National Hospital, P.O. Box 19676, Nairobi, Kenya; ^7^Department of Medicine, University of Washington, Seattle, WA 98195, USA; ^8^Department of Obstetrics, Gynecology and Reproductive Sciences, University of California, 50 Beale Street, Suite 1200, San Francisco, CA 94105, USA

## Abstract

*Study Objective*. To identify sensitive and specific histological criteria for endometritis in women with laparoscopically-confirmed acute salpingitis. *Methods*. Women, age 18–40 years of age presenting with complaints of lower abdominal pain ≤2 weeks and no antibiotics use in past two weeks, were enrolled. They underwent clinical examination, screening for HIV; other sexually transmitted infections plus endometrial biopsy sampling for histopathology. Diagnostic laparoscopy confirmed the diagnosis of acute salpingitis. Controls were women undergoing tubal ligation and HIV-1 infected women asymptomatic for genital tract infection. *Results*. Of 125 women with laparoscopically-confirmed salpingitis, 38% were HIV-1 seropositive. Nineteen HIV-1 negative controls were recruited. For the diagnosis of endometritis, ≥1 plasma cells (PC) and ≥3 polymorphonuclear lymphocytes (PMN) per HPF in the endometrium had a sensitivity of 74% for HIV-1-seropositive, 63% for HIV-1-seronegative women with a specificity of 75% and positive predictive value of 85% regardless of HIV-1-infection for predicting moderate to severe salpingitis. For HIV-1-seronegative women with mild salpingitis, ≥1 PC and ≥3 PMN had a sensitivity of 16% and a PPV of 57%. *Conclusion*. Endometrial histology, did not perform well as a surrogate marker for moderate to severe salpingitis, and failed as a surrogate marker for mild salpingitis.

## 1. Introduction

In Africa, pelvic inflammatory disease (PID) and its sequelae are a predominant cause of gynecologic morbidity [1, 2]. These include tubal factor infertility, ectopic pregnancy, chronic pelvic pain, and recurrent pelvic infections [3, 4]. 

HIV-1 seroprevalence in women with PID is consistently 2–7 times greater than measured in matched populations without PID [5–7]; both infections are most commonly acquired through unprotected sexual activity. Prompt diagnosis and treatment of women with upper genital tract infections is important in reducing morbidity, but it is complicated by lack of a sensitive and specific clinical and laboratory diagnostic test. Laparoscopy is the gold standard for the diagnosis of salpingitis, but is not practical for routine clinical practice.

Endometrial histopathology is often used as a surrogate for upper genital tract infection. Kiviat et al. [8] evaluated women with clinical PID; evidence of endometritis as defined by ≥1 plasma cell (PC) and ≥5 polymorphonuclear lymphocytes (PMN) per high-powered field (hpf) was 92% sensitive and 87% specific compared with visual findings of salpingitis determined by laparoscopy [8]. Using the same diagnostic criteria in a study of acute salpingitis in Kenya, plasma cell endometritis as defined by ≥1 PC/hpf was identified in 49% of women with salpingitis: this increased with disease severity and HIV-infection [7]. Studies on HIV-1-infected women have found an increased prevalence of plasma cell endometritis [9] even in the absence of clinical disease [10, 11].

Thus, we conducted this analysis to determine the optimum endometrial histopathological criteria for predicting salpingitis in a population with a high HIV-1 seroprevalence. We anticipate that these data will help to plan future clinical trials, increase the understanding of the pathogenesis of upper genital tract infection among HIV-1 infected women, and in certain circumstances provide a tool to confirm the clinical diagnosis of PID.

## 2. Materials and Methods

Study procedures have been previously detailed [12]. Briefly, between April 2000 and July 2002, women aged 18–40 admitted to Kenyatta National Hospital (KNH) acute gynecology ward with a complaint of lower abdominal/pelvic pain for 2 weeks or less plus one or more of the following signs or symptoms: temperature ≥ 38°C, dysuria, and complaint of abnormal vaginal discharge were eligible for enrollment.

After induction of anesthesia, an endometrial biopsy was obtained with a Pipelle suction curette (Unimar, Inc., Wilton, Conn). At laparoscopy, samples from peritoneal fluid, tubal ostia, and pyosalpinx/tubo-ovarian abscess (TOA) were obtained for *N. gonorrhoeae* and *C. trachomatis *PCR. Using the Jacobson and Westrom criteria [13], the severity of acute salpingitis was graded as (1) mild (tubal erythema or edema, mobile tubes, and with or without spontaneous exudate), (2) moderate (marked tubal erythema and edema, limited tubal mobility, questionable or no tubal patency, and gross exudate), and (3) severe (pyosalpinx or TOA). 

We enrolled two sets of controls. The first group included HIV-1-seronegative women presenting to the KNH family planning clinic. Women desiring permanent sterilization underwent laparoscopic tubal ligation preceded by an endometrial biopsy obtained using a Pipelle suction curette. HIV-1-seropositive controls were enrolled from an HIV care and treatment clinic at the Center for Respiratory Disease Research at the Kenya Medical Research Institute. Subjects had no clinical evidence of PID. Enrollment and study procedures of the HIV-1-seropositive control group are detailed elsewhere [14]. After informed consent was obtained, an endometrial pipelle biopsy was obtained in the research clinic.

### 2.1. Laboratory Methods

Samples from the cervix, endometrium, fallopian tube, and abscess were examined by PCR (Roche Molecular Diagnostics, Pleasanton, Calif, USA) for *N. gonorrhoeae *and* C. trachomatis*. Endometrial specimens were fixed in 10% buffered formalin, processed, and stained with hematoxylin, eosin, and methyl green pyronin. PMNs in glands and PCs in stroma were counted per high-power field. One pathologist (NK) who was blinded to the patients' diagnosis read the slides. Serum was tested for HIV antibodies by ELISA (Detect HIV, BioChem ImmunoSystems, Montreal, Canada) with positive results confirmed by a second ELISA (Recombigen, Cambridge Biotech, Ireland). 

### 2.2. Data Analysis

Data were analyzed using SPSS for Windows 10.0 (SPSS Inc., Chicago, USA). Univariate analyses used chi-square and Fisher's exact tests for categorical data and Student's *t*-test for continuous variables. Logistic regression was done for multivariate analysis.

## 3. Results

### 3.1. Description of Study Population

One hundred and sixty women were enrolled with clinical PID, 140 (88%) had laparoscopically confirmed salpingitis, 125 (89%) of whom had an endometrial biopsy specimen: 56 (45%) had mild, 31 (25%) had moderate, and 38 (30%) had severe disease based on laparoscopic criteria. Nineteen women had other diagnoses at laparoscopy including appendicular abscess (*n* = 2), endometriosis (*n* = 1), ovarian cyst (*n* = 12), frozen pelvis (*n* = 1), pelvic tuberculosis (*n* = 1), cancer of the sigmoid volvulus with abscess (*n* = 1), and ovarian torsion (*n* = 1). Asymptomatic women (*n* = 20) desiring permanent sterilization underwent laparoscopic tubal ligation and served as HIV-1-negative controls. A single control subject had a sticky exudate emanating from the Fallopian tubes and was excluded from the analysis leaving 19 HIV-1-seronegative controls. Forty-five asymptomatic HIV-1-seropositive controls were enrolled from an HIV care clinic; one woman had *C. trachomatis* detected.

Forty-eight (38%) of the women with salpingitis were HIV-seropositive. Women with salpingitis were younger, less likely to be married, and less likely to have ever used contraception (Table 1). As expected, none of the HIV-1-seronegative controls had signs or symptoms consistent with PID, and none were infected with *N. gonorrhoeae* or *C. trachomatis*. However, *T. vaginalis* was detected in a similar proportion of salpingitis cases (23%) and HIV-seronegative controls (21%) (Table 1).

### 3.2. Factors Associated with Evaluable and Unevaluable Endometrial Histopathology

Of the 125 women with salpingitis, endometrial biopsies from 107 (86%) were evaluated histological. Overall, 77 (72%) were adequate for histological diagnosis. Inadequate biopsies corresponded to endometrial specimens demonstrating sloughing, frank pus, and lack of tissue. In general, more severe disease as demonstrated by higher clinical severity score (CSS) (15.5 versus 13, *P* < .03) and severity of salpingitis based on laparoscopic findings (*P*-trend <  .04) was associated with unevaluable endometrial biopsy results (Table 2). Similarly, history of depomedroxyprogesterone acetate (DMPA) was associated with an increased likelihood of obtaining an unevaluable biopsy (*P* = .02). HIV-infected women were more likely to have an unevaluable endometrial biopsy (57% versus 36%, *P* < .05) than HIV-uninfected women. Although not significant, participants with an inadequate endometrial histological specimen had a higher prevalence of gonorrhea compared to those with an adequate biopsy (23% versus 12%  *P* < 0.23) (Table 2).

In multivariate analysis, after controlling for factors found significant in univariate analysis, the use of DMPA at any time (adjusted OR = 3.1, 95% CI 1.1–8.5), HIV-1 infection for women with mild (AOR = 4.6, 95% CI 1.1–18.3) but not moderate salpingitis (AOR = 0.89, CI 0.15–5.3), or severe salpingitis (AOR = 2.63, CI 0.68–10.2) was associated with an increased odds of an unevaluable endometrial biopsy. In addition, 12 (63%) of 19 specimens from HIV-1-seronegative subjects were evaluable for histopathology.

### 3.3. Distribution of PMN and PC in the Endometrial Biopsy: Effect of HIV-1 Serostatus and Disease Severity

We reviewed the distribution of PMN and PC by HIV-1 serostatus and severity of salpingitis. Women with severe salpingitis regardless of HIV-1 serostatus had the highest frequency of PMN and PC per high-power field. Only two patients with HIV-1 infection and salpingitis did not have PMN found in the endometrium. Although PMN density did not increase with severity of salpingitis among women with HIV-1 infection (*P*-trend = .49), this association was significant for HIV-1-uninfected women with salpingitis (*P*-trend = .05). In contrast, the frequency of PCs increased with severity of salpingitis among those with HIV-1 infection (*P*-trend = .04), but not among HIV-1 uninfected (*P*-trend = .14). Furthermore, HIV-1 infection was associated with a higher frequency of PCs/hpf (*P*-trend <.001), and presence of lymphoid follicles (*P* < .04). Only 2 (6%) of 34 HIV-1-infected women with salpingitis did not have any plasma cells present in the endometrium versus 23 (41%) of 56 HIV-1-uninfected women with salpingitis.

### 3.4. Comparison of Endometrial Histopathology Findings and Salpingitis

We next set out to determine the sensitivity, specificity, and positive predictive value of four histopathologic criteria for diagnosis of endometritis in comparison to the laparoscopic diagnosis of salpingitis. The four rules evaluated included: (a) ≥3 PMN and ≥1 PC per high-power field, (b) ≥1 PMN and ≥1 PC per high-power field, (c) ≥1 PMN per high-power field, and (d) ≥1 PC per high-power field. Women with moderate and severe disease were grouped together and compared to women with mild salpingitis and to the two control groups. Table 3 outlines the comparison between the laparoscopic diagnosis for mild and moderate/severe salpingitis and the four histological rules stratified by HIV-1 serostatus. Because the diagnosis of the moderate and severe disease requires more objective evidence of tubal inflammation (e.g., pus from tubes, pyosalpinx, abscess, and fresh adhesions) than mild disease, we chose to gauge the sensitivity of each histological rule using laparoscopic evidence of moderate/severe salpingitis as the “gold standard.” Rule “a”, although less sensitive than rules “b” through ”d” for women with moderate/severe salpingitis (HIV-seropositive = 74% versus 63%; HIV-seronegative; 93% versus 75%), was the most specific, demonstrating endometritis in 25% and 7% of HIV-1-seronegative and HIV-1-seropositive controls, respectively, in comparison to 58%–67% and 38%–62% for rules “b” through “d”. Among the 19 women enrolled with a clinical diagnosis of PID, but who did not have salpingitis on laparoscopy, rule “a” had the least false positive, while rule “d”, at least one plasma cell, scored the highest false-positive rate.

## 4. Discussion

This study had three key findings: (1) ≥3 PMN and ≥1 PC per hpf as a histologic criteria for the diagnosis of moderate to severe salpingitis, while performing better than the other criteria, appears to have limited utility even more so for cases of mild salpingitis; (2) endometrial specimens were often unevaluable for histopathology, and unevaluable specimens were more likely in subjects with severe salpingitis and HIV-infection, and thus may affect the utility of endometrial histopathology to confirm the clinical diagnosis of PID in similar settings; (3) The PMN response increased with disease severity for HIV-1 seronegative but not HIV-1 seropositive women with salpingitis.

Since Kiviat et al. published their paper, [8] histologic endometritis has been used as a surrogate marker for salpingitis, especially in the study of mild to moderate PID. Even though the criteria for histologic endometritis had never been validated in HIV-1-infected populations, several studies of PID were conducted in high HIV-1 seroprevalence settings [6, 7, 9, 12, 14]. The results of this study did not validate the Kiviat et al. 1990, criteria for ≥5 PMNs and ≥1 PC for the diagnosis of PID. In the Kiviat et al. cohort, *N. gonorrhoeae* and/or *C. trachomatis* was found in 49% of the patient population; in comparison, the cohort in our study had a high HIV-1 prevalence and a combined gonorrhea and/or chlamydia prevalence of 18% (Table 1). 

Similar to another report, only 72% of endometrial biopsies in our study were evaluable [1]. The increased frequency of unevaluable endometrial biopsies in women with severe salpingitis, likely due to increased endometrial sloughing and presence of pus, and HIV-1 infection further limits the utility of endometrial histopathology as a diagnostic tool for studies of PID in similar populations. 

The low sensitivity of histologic endometritis for mild salpingitis amongst women symptomatic for PID was unexpected. Studies of endometritis in populations of asymptomatic women have consistently demonstrated a relatively high prevalence of endometritis [10, 15, 16] which led authors to describe endometritis as an intermediate infection to PID. Eckert et al. studied HIV-1-infected women presenting to a family planning clinic and found endometritis in 38% of participants [10]. This is a higher prevalence than what we found in HIV-1-negative women (16%) and a lower prevalence than what we found in HIV-1-seropositive women (57%) with mild salpingitis using less stringent criteria for endometritis. Furthermore, a prior laparoscopic study demonstrated salpingitis in the absence of endometritis [7]. An alternative explanation may result from the subjectivity of the laparoscopic criteria for mild salpingitis that leads to misclassification of cases [13]. 

The distribution of PMNs and PCs in the endometrium of women with salpingitis was affected by HIV-1 serostatus and disease severity. PMNs are only found in the healthy endometrium during menses [17], and form part of the endometrial immune response, they are also the first line immune defense against bacterial infections. The increased density of PMN with severe disease in HIV-1-uninfected but not in HIV-1-infected women with salpingitis is not well understood. Consistent with other studies [7, 9, 10, 14], we found increased PC endometritis with HIV-1 infection. This could represent HIV-1 infection in the genital tract [18]; chronic plasma cell endometritis [11, 14]; or the presence of opportunistic infections. Cherpes et al. reported an association between HSV-2 seropositivity and plasma cell endometritis [19]; notably HSV-2 is extremely prevalent among HIV-1-infected persons (KAIS 2007 [20]. Contrary to these findings, Eckert et al. [10] evaluated 20 endometrial biopsy samples from women with asymptomatic histologic endometritis and failed to detect herpes simplex virus by PCR, and cytomegalovirus was detected equally in women with and without histological endometritis. *Mycoplasma genitalium* is another potential cause of endometritis [21]. 

One limitation of this study is that HIV-1-infected controls did not undergo laparoscopic evaluation. Therefore, unlikely we cannot firmly exclude subclinical salpingitis from this population as we can for the HIV-1-seronegative controls. Furthermore, we did not attempt to detect suspected etiologies of endometritis such as bacteria other than *N. gonorrhoeae* and *C. trachomatis* including *M. genitalium* [14] and potential etiologies such as cytomegalovirus and herpes simplex virus infection. Such data might help to elucidate the reason for the different findings among HIV-1-seropositive and HIV-1-seronegative women with salpingitis in regards to endometrial histopathology.

This study raises some important questions regarding PID and its sequelae. With increased access to highly active antiretroviral therapy (HAART), HIV-1-infected women are living longer. Population data from Uganda [22] plus others [23] have demonstrated reduced fertility in HIV-1-infected women regardless of disease stage. It is plausible that PC endometritis may lead to reduced fertility. Further research is required to determine if women using HAART return to normal fertility or not. Lastly, although endometrial histopathology serves as a reasonable surrogate for salpingitis in HIV-1-uninfected populations, its utility in populations with a high HIV-1 seroprevalence appears to be limited. Discovery of a sensitive and specific biomarker or set of biomarkers for salpingitis could facilitate further research on PID and its sequelae in such settings. 

## Figures and Tables

**Table 1:** Comparison of demographic, clinical history and signs, and laboratory findings for women laparoscopically diagnosed with salpingitis and women undergoing tubal ligation (controls).

Variables	Salpingitis	HIV −/ve Controls	*P* value
	*N* = 125	*N* = 19	
*Demographics and clinical history*			
Age mean years (SD)	27.8 (5.5)	34.3 (4.1)	0.001
Education mean years (SD)	8.7 (2.9)	8.2 (2.5)	0.5
Marital status:			
Single	31 (25%)	0	Ref.
Married	70 (56%)	17 (90%)	0.008
Divorced/separated	20 (16%)	2 (10%)	0.12
Ever use of contraceptives:			
Oral contraceptives	55 (44%)	16 (84%)	0.001
DMPA	40 (32%)	15 (80%)	0.001
IUD	19 (15%)	8 (42%)	0.005
Condoms	48 (39%)	3 (16%)	0.04
*Clinical findings*			
Clinical severity score (CSS), median, (mode), range	14 (8) 32	0 (0) 5	0.00
*Laboratory findings*			
HIV-1	48 (38%)	0	0.001
Gonorrhea and/or chlamydia	23 (18.4)	0	0.04
*Trichomonas vaginalis*	23 (19%)	4 (21%)	0.8
Adequate endometrial biopsy	77 (72%)	12 (63%)	0.4

**Table 2:** Comparison of demographic, clinical history and signs, and laboratory findings for women laparoscopically diagnosed with salpingitis, with and without an endometrial biopsy adequate for histological evaluation.

Variables	Adequate biopsy	Inadequate biopsy	*P* value
	*N* = 77 (72%)	*N* = 30 (28%)	
*Demographics and history*			
Age mean (SD)	27.8 (5.5)	27.9 (6.1)	0.9
Infertility ≥ 1 year	26 (39%)	3 (13%)	0.02
Ever use of contraceptive:			
None	56 (51%)	23 (77%)	Ref
Oral contraceptives	36 (47%)	13 (43%)	0.75
DMPA	18 (23%)	14 (47%)	0.02
Intrauterine device	13 (17%)	3 (10%)	0.37
*Symptoms*			
Abnormal menstruation	16 (21%)	6 (21%)	1.0
*Clinical examination findings*			
Clinical severity score, median (mode) range			
Total clinical severity score	13 (8) 28	15.5 (4) 30	0.03
Laparoscopic salpingitis severity			
Mild	32 (42%)	15 (50%)	
Moderate	26 (36%)	3 (10%)	
Severe	19 (25%)	12 (40%)	0.04
Pelvic Abscess	13 (17%)	9 (30%)	0.15
*Laboratory Findings*			
HIV-1	27 (36%)	17 (57%)	0.05
CD4 count < 200/*μ*L	8 (11%)	7 (23.3%)	0.09
White cell count	9.7 (6.04)	10.3 (5.5)	0.63
Lymphocytes (blood) %	26.9 (13.4)	19.1 (7.8)	0.001
Gonorrhea and/or chlamydia	11 (14%)	8 (27%)	0.13
Gonorrhea	9 (12%)	7 (23%)	0.23
Chlamydia	3 (4%)	1 (3%)	0.9
*Trichomonas vaginalis*	14 (19%)	6 (21%)	0.8
Bacterial vaginosis (Gram's stain)	30 (45%)	16 (62%)	0.2

**Table 3:** Presence and density of polymorphonuclear leucocytes (PMN) and plasma cells (PC) on histopathology of endometrial biopsies in women with mild, moderate, and severe salpingitis at laparoscopy and controls in HIV-seropositive and -seronegative women.

Rules representing cell/hpf	Mild salpingitis		Moderate/severe Salpingitis		Controls without salpingitis	
	*N* = 32		*N* = 50		*N* = 62	
	HIV +/ve	HIV −/ve	HIV +/ve	HIV −/ve	HIV +/ve	HIV −/ve
	(*N* = 7)	(*N* = 25)	(*N* = 23)	(*N* = 27)	(*N* = 45)	(*N* = 12)
≥3 PMN and ≥1 PC	4 (57%)	4 (16%)	17 (74%)	17 (63%)	3 (7%)	3 (25%)
≥1 PMN and ≥1 PC	7 (100%)	8 (32%)	19 (83%)	20 (74%)	17 (38%)	7 (58%)
≥1 PMN	7 (100%)	17 (68%)	21 (91%)	21 (78%)	32 (71%)	8 (67%)
≥1 PC	7 (100%)	9 (36%)	21 (91%)	21 (78%)	28 (62%)	8 (67%)
